# Prediction Methods in Solar Sunspots Cycles

**DOI:** 10.1038/srep21028

**Published:** 2016-02-12

**Authors:** Kim Kwee Ng

**Affiliations:** 1 Dept of Physics and Astronomy, State University of New York at Stony Brook, NY 11794, USA.

## Abstract

An understanding of the Ohl’s Precursor Method, which is used to predict the upcoming sunspots activity, is presented by employing a simplified movable divided-blocks diagram. Using a new approach, the total number of sunspots in a solar cycle and the maximum averaged monthly sunspots number *Rz*(max) are both shown to be statistically related to the geomagnetic activity index in the prior solar cycle. The correlation factors are significant and they are respectively found to be 0.91 ± 0.13 and 0.85 ± 0.17. The projected result is consistent with the current observation of solar cycle 24 which appears to have attained at least *Rz*(max) at 78.7 ± 11.7 in March 2014. Moreover, in a statistical study of the time-delayed solar events, the average time between the peak in the monthly geomagnetic index and the peak in the monthly sunspots numbers in the succeeding ascending phase of the sunspot activity is found to be 57.6 ± 3.1 months. The statistically determined time-delayed interval confirms earlier observational results by others that the Sun’s electromagnetic dipole is moving toward the Sun’s Equator during a solar cycle.

The Sun is an important source of energy in our life. The sunspots and solar flares have been observed and studied by many researchers. Many models which are developed from the Ohl’s Precursor Method^1^ have yielded varying degrees of success in predicting an upcoming solar activity. In this study, a simplified movable divided-blocks diagram is proposed to explain the underlying physical principle of the Ohl’s Precursor Method. Based on the movable divided-blocks diagram, the most promising and interesting regions for statistical evaluation are identified. The strong correlationship between the sunspots activity numbers and the geomagnetic index is numerically calculated and subsequently verified.

It is consequently shown from a statistical study of the time-delayed solar events that the electromagnetic pole members to which the sunspots are associated, are moving with a time interval which is determined statistically in this study, toward the Sun’s Equator in each solar cycle under the influence of electromagnetic torques which are the higher-orders corrections to the rotating magnetic dipoles.

The sunspots, though seem to have occurred randomly on the Sun, have exhibited another regular feature. A butterfly pattern, which is observed in each of the solar cycles over the centuries, has long been a puzzling phenomenon. The butterfly pattern, as shown below, could be inferred from the various projected end-points left by the precessing motion of the magnetic moments under the influence of the electromagnetic torques.

The calculated results are compared with the observed sunspot butterfly diagram. The similarity between them is interesting for further investigation and discussion. A recent attention has been focusing on the possible forthcoming new ice-age like climate^2^, which is based on a mathematical regression model that extracts two principal components in the solar background magnetic field. This effect would have a serious impact in our life. An understanding of the sunspots activity and a long-term planning would help us to better prepare with the changes.

The correlation factors are calculated from the solar and geomagnetic data sets with the selected time-windows as implied and chosen from the movable divided-blocks diagram. A statistical study of the time-delayed events, which would eventually produce a reasonable prediction on the timing of the peak in the monthly sunspots numbers in the ascending phase of an upcoming solar cycle, is presented for further investigation.

## Results

### Results from the Observation

Smith^3^ *et al.* have found from a reconstructed three-dimensional heliosphere at the maximum of a solar activity that the heliospheric magnetic field originating from a Sun’s magnetic dipole is oriented almost perpendicular to the Sun’s rotation axis. The Sun’s magnetic dipole, according to their reconstruction, is rotated from its initial axial position and oriented gradually toward its opposite heliographic pole^3^. The rotational movement of the Sun’s magnetic dipole, as interpreted in their analysis in a phenomenon called a magnetic pole reversal, is consistent with the trajectory motion^4^ of the magnetic moment under the action of an electromagnetic torque *τ*_*y*_ of Equation (2) to be shown below.

Furthermore, they have suggested that the changes in the solar magnetic field is inconsistent with a simple rotating dipole^3^. Multiple sets of magnetic moments, moving in different groups, are employed in our study as explained in the sections below.

The description of the rotation of the Sun’s magnetic dipole as stated above paves the way for an in-depth understanding of the physical reason underlying the Ohl’s Precursor Method^1^. A divided-blocks diagram is employed to explain the relationship found between the observed geomagnetic signals and the maximum averaged monthly sunspots number *Rz*(max) to be seen in the next solar cycle.

### The Physical Theory on a Rotating Magnetic Moment

**Figure 1:**
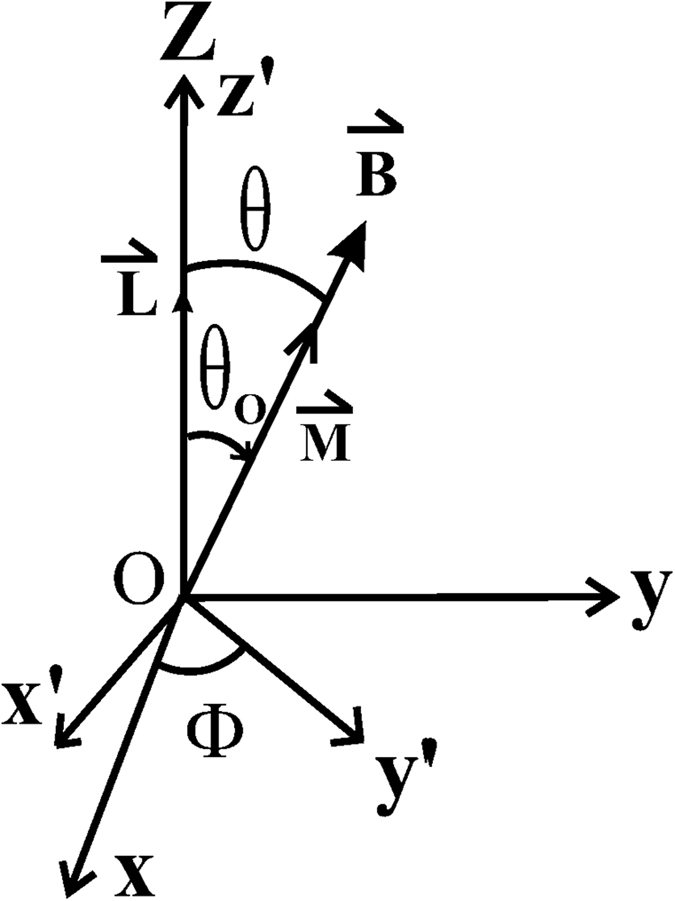
The magnetic moment M in a *xyz* coordinate system.

The theory on a rotating magnetic body has been developed over the decades. Deutsch^5^ considers a magnetized star having a magnetic moment M and an angular frequency Ω spinning in a vacuum as shown in Fig. 1. He has found a spin-down effect for the spinning star. Other electromagnetic torques have since been found^6^,^7^,^8^ and studied^9^,^10^,^11^. A different approach is to study the effect of a retarded magnetic field caused by a primary current density J_*p*_ flowing with a radius *a*, with the current density as 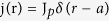. It is subsequently expressed in terms of the familiar magnetic moment M we are accustomed to by using 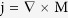. The current density j(r, *t*) at different points and time *t* is expanded into a power series consisting of a series of power factors of *v*/*c*, where *v* = *r*Ω. The electromagnetic torques in a Cartesian (xyz) coordinate system are listed below in Equations (1, 2, 3) 12.


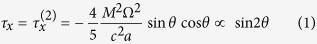



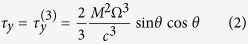



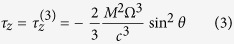


The boundary condition introduced by Deutsch^5^ for solving the Maxwell Equations, namely the star’s conducting surface employed in his simplified equations, introduces only a small difference in the near-field effect. The numerical coefficient calculated^6^ from Deutsch’s equation is 1 in the conducting case^5^, rather than the numerical factor 4/5^12^ as indicated in Equation (1). A study of the effects from different distribution of charges and current density has recently been attempted^11^.

### Comparison of the Theory with the Observational Results

The spin-down effect due to the z-component *τ*_*z*_ of the electromagnetic torque has been verified through the observation of pulsars. The observed spin-down is often viewed as a consequence resulting from the magnetic dipole radiation in many literature^13^. Stairs^14^ *et al.* have made a discovery that the electromagnetic signals arriving from the pulsar PSR B1828–11 are highly periodic. The variations in the lighthouse-like beams of radio signals are correlated in both the pulse shape and the rate of spin-down of the pulsar. They have presented a strong evidence that a precession has occurred in the pulsar. It is found that the unique signature in the radio signals on the precession can best be explained by two precessing magnetic moments in the pulsar^4^ when the x-component *τ*_*x*_ of the electromagnetic torque described above is included.

The second-order electromagnetic torque *τ*_*x*_ of Equation (1) causes the magnetic moment M to undergo a 2-dimensional precessing motion. The third-order electromagnetic torque *τ*_*y*_ of Equation (2) produces a slightly complicated 3-dimensional motion that leads eventually to a magnetic pole reversal. The reversal motion is shown in detail in the ref. 4.

It would be shown below that a butterfly pattern is formed by a group of electromagnetic pole members which have left their marks in the Sun during a solar cycle. These happen when the magnetic moments are momentarily at rest at one of their two instantaneous terminating end-points during each cycle of the precessing motion of the magnetic moments under the actions of the second-order and the third-order correction terms of the electromagnetic torques as explained above.

### Efforts and works on predicted sunspot numbers

The sunspot Wolf number, *Rz*, also called relative sunspot number, is an useful index to measure the sunspots activity. The daily sunspot Wolf number, *Rz*, is calculated from the total number of individual sunspots and the observed number of sunspot groups. It is based on a formula, *Rz* = *k*(10*g* + *s*), where *s* is the number of individual spots, *g* is the number of sunspot groups, and k is an adjusted localized observation factor. The geomagnetic activity index *aa* is a measure of the magnetic disturbances at an invariant magnetic latitude of 50 degrees. Ohl^1^ has proposed to use the geomagnetic activity index *aa* to predict the maximum averaged sunspot Wolf number *Rz*(max) in the succeeding solar cycle. Other methods based on the same principle but varying in their approaches are Thompson’s Method^15^ and Feynman-based method^16^. The principle underlying these precursor methods is not well understood. In the following section, a simplified movable divided-blocks diagram is presented to explain the relationship between the geomagnetic signals received by the Earth and a group of active electromagnetic pole members which would eventually appear to form a butterfly diagram near the Sun’s Equator.

It is found that the cumulative sum of the geomagnetic activity index *aa* in a 2-years period prior to the beginning of a solar cycle and the total number of sunspots in the succeeding solar cycle are well correlated. Moreover, the peaks of the geomagnetic activity index *aa* in the preceding solar cycle are also found to be correlated to the maximum averaged monthly sunspots number *Rz*(max).

Based on an analysis to be shown below, two methods are proposed to predict the maximum averaged monthly sunspots number *Rz*(max) in the upcoming solar cycle. A statistical study of the sunspots numbers, to be discussed in detail in the following sections, has found that the current solar cycle 24 is similar in some aspects to the solar cycle 16. The sunspots activities in the current solar cycle 24 are well below what we had in the previous few solar cycles. Pesnell^17^ recently uses a geomagnetic precursor pair to predict an amplitude of 65 ± 20 in the smoothed sunspots number for solar cycle 24. Kane^18^ uses 12-month running averages of the geomagnetic activity index *aa* to predict the maximum averaged monthly sunspots number *Rz*(max) for the current solar cycle 24. Kane’s predicted number was 58 ± 25.0. Bhatt^19^ *et al.* have found the number to be 92.8 ± 19.6.

A consensus statement on solar cycle was released prior to solar cycle 24^20^. A Combined Precursor Method which uses the weighted averages from the Thompson’s Method^15^ and the Feynman’s Method^16^ has produced a better predictive power on the solar activity^21^. Other related works over the decades have also been made^22^,^23^. An alternative way to predict the solar activity is a regression method which produces a month-by-month estimate of the solar cycle’s activity level^21^. Past predictions on earlier solar cycles have also been made by other investigators^24^,^25^.

Other investigators have used the strength of the polar magnetic fields to estimate the amplitude of an upcoming solar cycle^17^. The predicted numbers are 75 ± 8^26^ and 80 ± 20^27^ respectively. It is noted that the magnetic pole members at high latitude of the Sun, as projected by the magnetic moments, are similar in the description to the polar magnetic fields reported in the literature. It has been observed that the electromagnetic activity at latitudes above those populated by many active sunspots of the current cycle may be the source which gives rise to the precursor disturbances^21^. The electromagnetic signals emitted at the Sun’s high latitudes are received and recorded as the observed geomagnetic index aa by the Earth’s recording stations.

It would be shown below that the observation on the source of the electromagnetic disturbances from the Sun’s higher latitude, is consistent with the movable divided-blocks diagram proposed in the following section.

### Statistical Evaluation: the basis of the evaluation

A butterfly pattern, which resembles a butterfly in a Sun’s time-latitude plot^28^ when the sunspots appear, has a cycle of about 11–12 years. The cycle, known as a solar cycle, is also related to a cyclic process called magnetic poles reversal in which the polarity of the Sun’s magnetic dipole is reversed.

The fact that the period of a butterfly cycle is the same as the period of a magnetic pole reversal has important physical significance and implication on the rotational movement of the magnetic dipole moments. Based on the trajectories made by the apparent migration paths of the sunspots that eventually produce the butterfly patterns over the years, and together with the knowledge that the sunspots are strongly associated with the magnetic field, it would be shown in the following sections that the rotational movements of the magnetic dipole moments have played an important role in the formation of the butterfly patterns which are assembled from the apparent locations of the sunspots during the solar cycles.

**Figure 2:**
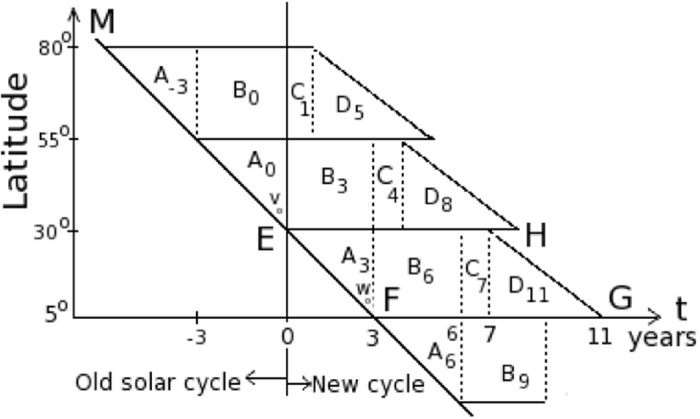
Simplified movable divided-blocks butterfly diagram.

**Figure 3:**
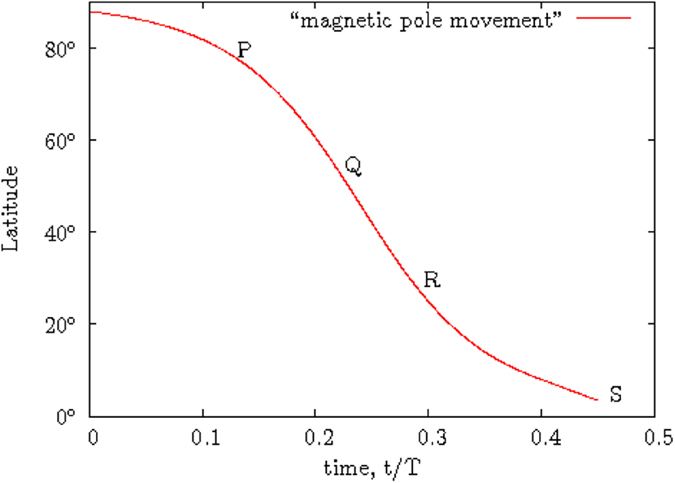
Movement of a magnetic pole from P to S.

The following example is a simple illustration of the movable divided-blocks diagram on how a butterfly diagram EFGH (approximated to represent the butterfly diagram seen during a typical solar cycle) would be subsequently formed from the various moving blocks in Fig. 2. For simplicity, some simple numbers are used in the following illustration. A typical solar cycle is 11 years. The duration in the ascending phase in the butterfly diagram is three years. The butterfly diagram EFGH shown in Fig. 2 comprises of four blocks, *A*_*i*_, *B*_*j*_, *C*_*k*_ and *D*_*l*_, where *i*, *j*, *k*, *l* are the time indices (years) when the named block *A*, *B*, *C* or *D* has reached its position shown in Fig. 2. Each individual block is sliding along a line parallel to the line MEF, which corresponds to the time-line curved track PQRS shown in Fig. 3.

Figure 3 is the perceived movement of the electromagnetic pole members during the first half of a solar cycle under the third-order correction term of the electromagnetic torque *τ*_*y*_. The blocks A, B, C and D in Fig. 2 are not actually moving at a constant speed when the blocks are sliding down the time-line curved track PQRS of Fig. 3. However, a constant moving speed is substituted for easy understanding of the complex picture involved in the movement of the active members contained in the various blocks A, B, C and D. The shapes of the blocks are in fact not well defined during the block movement from a point P to the point S in Fig. 3.

At time *t* = 0, the blocks *A*_o_ and *B*_o_ appear in the high latitude from 30° to 80°. As the time *t* increases, the blocks *A*_o_ and *B*_o_ slide downward along the line MEF. At *t* = 3 years, the blocks *A*_o_ and *B*_o_, which are now labeled respectively as *A*_3_ and *B*_3_, have reached a new position as shown in Fig. 2. The block *A*_3_ has become the ascending phase of the butterfly diagram EFGH. As the time *t* continues to increase, *A*_3_ would become *A*_6_ at *t* = 6 years. The block *B*_3_ would become *B*_6_ in the new position in Fig. 2. The blocks *A*_3_ and *B*_6_ become the first half of the butterfly diagram EFGH.

A new solar cycle begins at *t* > 0, and it is characterized by the first appearance of a positive or negative polarity-leading sunspot in the high latitude, opposite in polarity to the previous solar cycle. This is shown as a point v in the block *A*_o_, as the point v slides along the line MEF.

The electromagnetic signals emitted by the participating members in the blocks *A*_o_, *B*_o_, and the members in other partially appearing blocks *C* and *D* (not shown) at high latitude of the Sun are the precursor activity indicators for the upcoming solar cycle. The strength of the electromagnetic signals, including signals from other extraterrestrial events received by the Earth, are recorded as the geomagnetic activity index, now commonly labeled as *aa*. The index *aa* is also impacted by the activity from the sunspots and solar flares. The Sun is typically in a relatively quiet period near the end of a solar cycle. Thus, the geomagnetic activity index *aa* in the late declining phase of the solar cycle can be used as a good indicator of the upcoming sunspots activities in the new solar cycle. The illustration using the movable divided-blocks as shown in Fig. 2 is supported and verified in the data analysis shown below. Furthermore, a practical application in the use of the proposed movable divided-blocks diagram would be illustrated in the time-delayed solar events below.

### Statistical Results: Graphs and Correlation Factors

The solar butterfly diagrams observed throughout the centuries have implied that the process involving the sunspots and the magnetic poles reversals takes an extended time to complete. Hence, it would be advantageous to consider a time division in a block diagram, such as the proposed block diagram described above, to easily understand and visualize the dynamic picture of the process involved.

**Figure 4:**
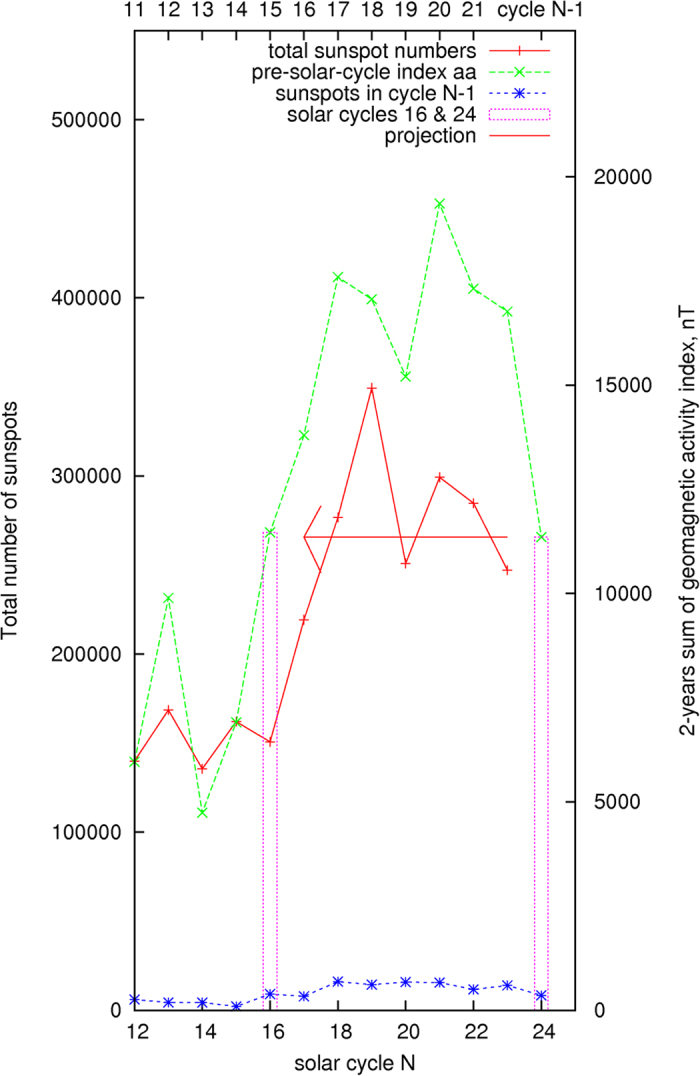
Geomagnetic activity index and total number of sunspots in solar cycle N.

**Figure 5:**
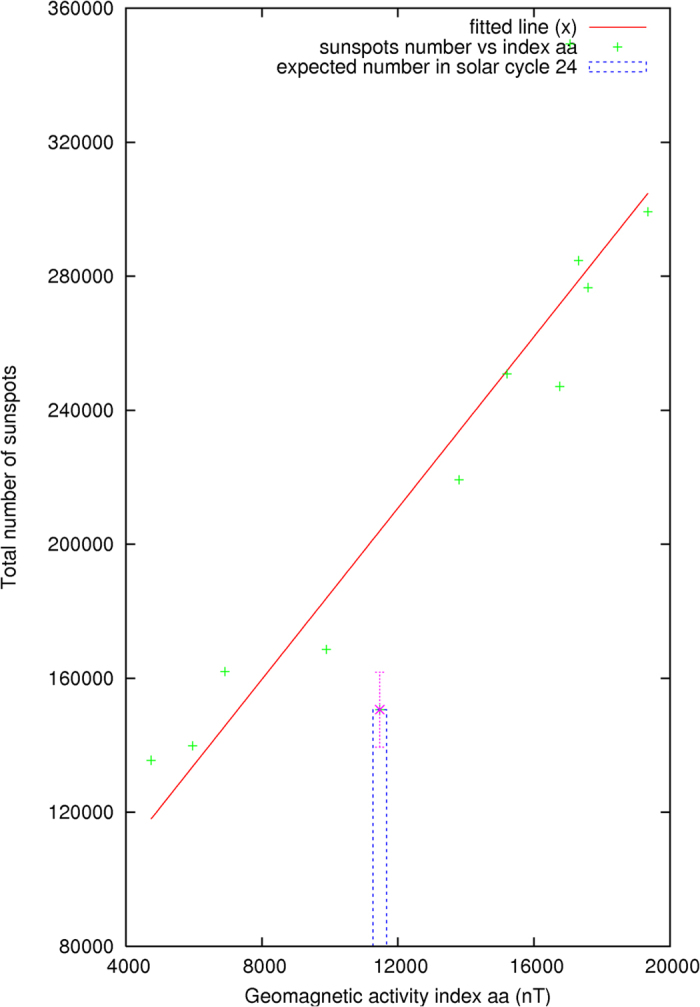
Correlation of activity index and total number of sunspots.

As explained in the illustration above in Fig. 2, the daily averages of the geomagnetic activity index *aa* in the two years preceding the end of each solar cycle are added. The rationale for the selected time-window interval of 2–3 years and the summation are motivated and implied from the movable divided-blocks diagram shown in Fig. 2. The summation of the activity index *aa* is compared with the total number of sunspots in the corresponding succeeding solar cycle N. The results are shown in Fig. 4. Their correlation factor, calculated and shown in Fig. 5, is 0.91 ± 0.13. The t-value representing the statistical significance of the result is 10.9, df = 11. The p-value is <0.0001, or ~5*σ* (Fig. 5). The result is said to be statistically very significant.

In Fig. 4, the number of sunspots in the prior cycle (during the same 2-years period in solar cycle *N* − 1) is also shown. The sunspots occurring in the previous solar cycle contribute as a background noise to the values of the geomagnetic activity index *aa*. The magnitude of their impacts to the values of the geomagnetic activity index *aa* is subject to further investigation^16^,^21^. The background noises would be higher if the above selected time interval is expanded from 24 months to 30 months and 36 months, the fact on their strong correlationship has however remained unaffected at 0.92 ± 0.12, 0.93 ± 0.11 respectively.

**Figure 6:**
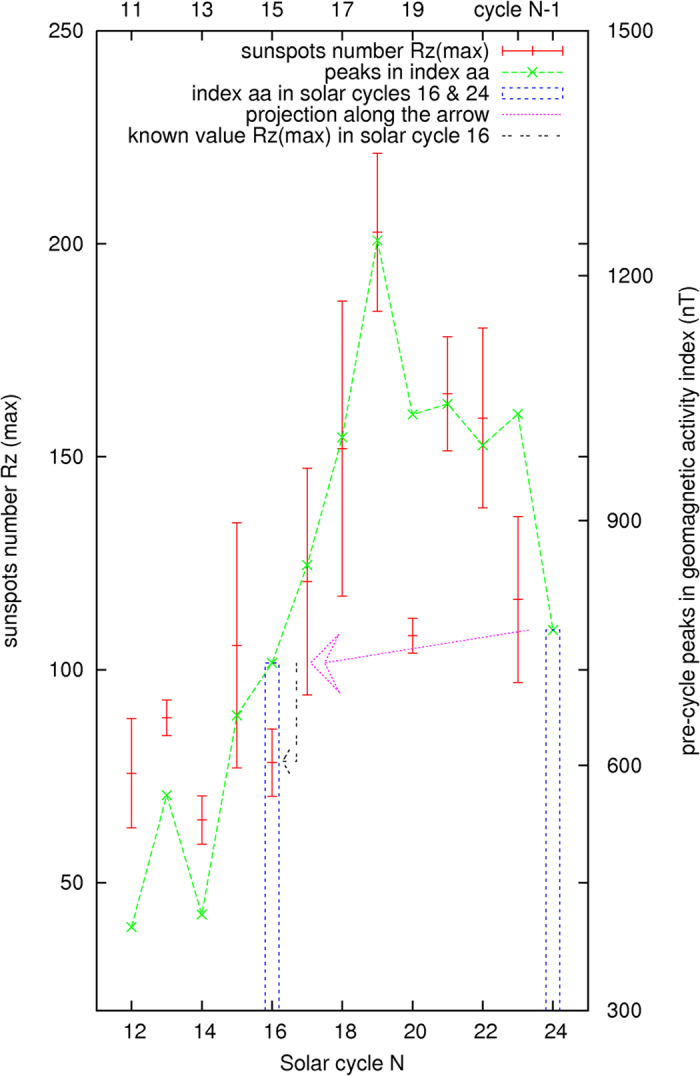
Pre-cycle peaks found in the activity index and sunspot number *Rz*(max) in solar cycle N.

**Figure 7:**
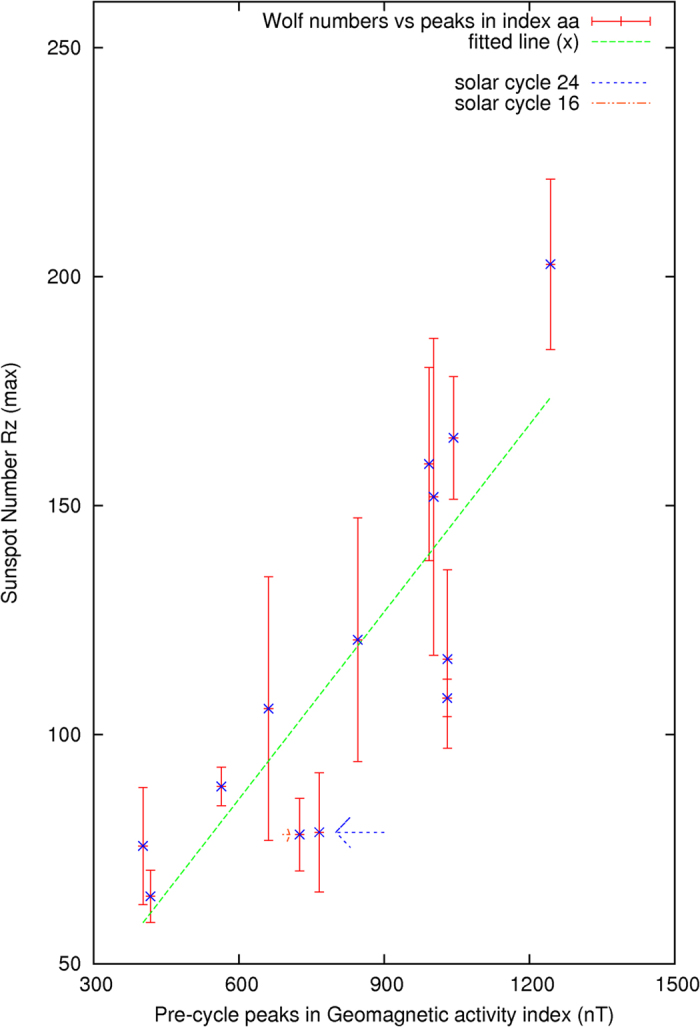
Correlation of the peaks in activity index and sunspot number *Rz*(max).

Furthermore, the first prominent peak found in the monthly geomagnetic activity index *aa* in the prior 30 months preceding the end of each solar cycle is compared with the maximum averaged monthly sunspots number *Rz*(max) in the succeeding solar cycle. The monthly sunspots number *Rz* is a monthly number of the sunspots averaged over a 12-month period in the solar cycle. An extremely narrow and enormously high value of the index *aa* occurring between May 14 and May 16, 1921 (Δ*aa* ~ 530) is corrected from the analysis. The abrupt change in the index *aa* was likely caused by some other mysterious events. The results are shown in Fig. 6. The correlation factor is 0.85 ± 0.17. They are statistically correlated as shown in Fig. 7. The t-value representing the statistical significance is 11.3, df = 12. The p-value is <0.0001, or ~5*σ* (Fig. 7). The result is considered to be statistically very significant.

Based on the information given by Fig. 4, as shown by a horizontally pointing arrow, the current solar cycle 24 seems to be similar to the solar cycle 16 of the Years 1923–1933, which has a maximum averaged monthly sunspots number *Rz*(max) at 78.2 ± 7.9. The arrows in Fig. 6 show a similar trend, the maximum averaged monthly sunspots number *Rz*(max) of the current solar cycle could be estimated to have a range centered at about ~80 ± 10, which is derived from the solar cycle 16 and the data in Fig. 6, in addition to the use of the results from Fig. 4.

The error bars in Figs 6 and 7 are calculated to be 1*σ*, one standard deviation away from the averaged monthly sunspots number *Rz*, which is evaluated as the 12-month running average of the observed monthly sunspots numbers.

**Figure 8:**
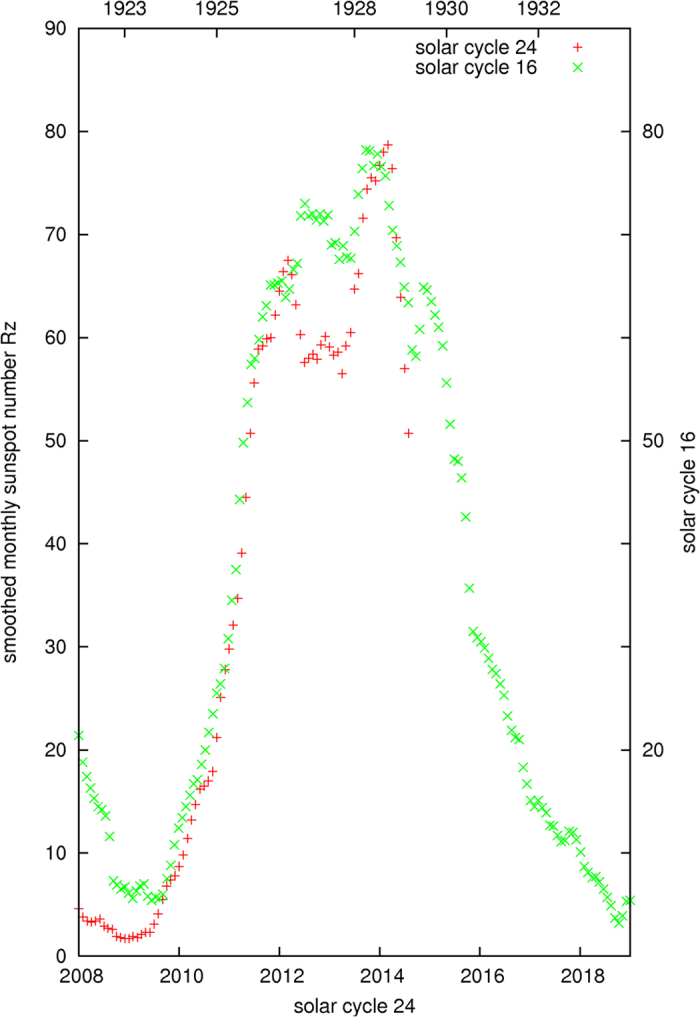
Similarity between the solar cycles 16 and 24.

The preliminary maximum averaged monthly sunspots number *Rz*(max) for the current solar cycle 24 now appears to be 78.7 ± 11.7 in March 2014. As illustrated above, the calculated results from the two methods, as determined from Figs 4 and 6, are consistent with the currently observed solar cycle 24. The total number of sunspots in the current solar cycle 24 is estimated to be 150,000 ± 11,000 as shown by a vertical bar in Fig. 5. The similarity, but not identical, between the solar cycles 16 and 24 is noted in Fig. 8, where their 12-month moving averaged, smoothed monthly sunspots numbers *Rz* are respectively shown in the diagram.

**Figure 9:**
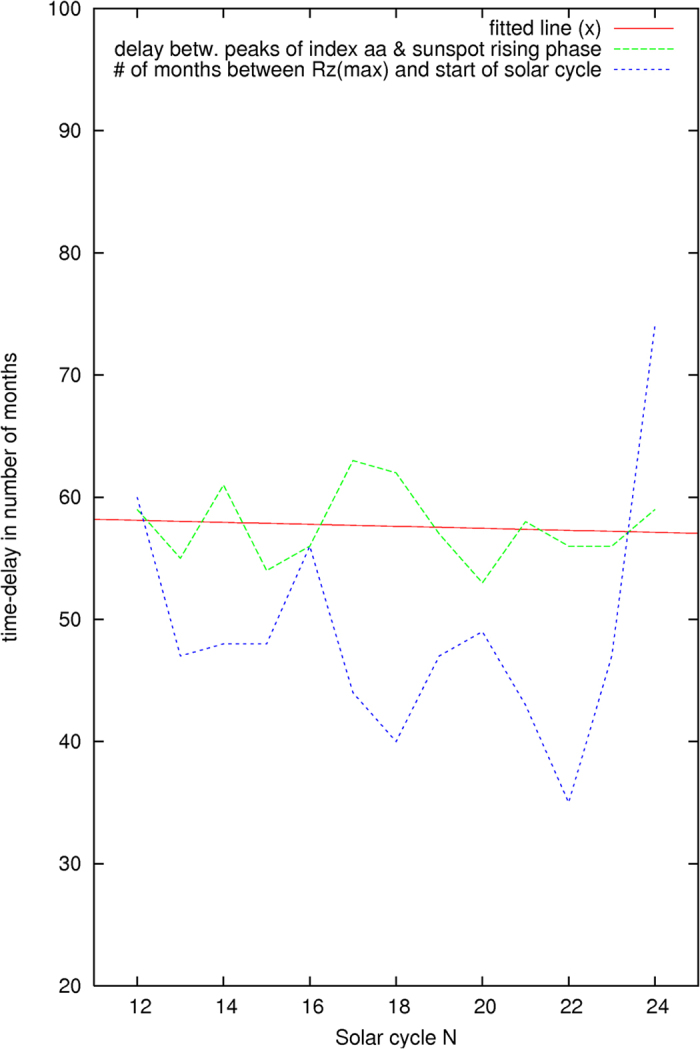
Delay time found between the peaks in the solar events.

Figure 9 illustrates the time delay between the solar events. The first curve is the number of months between the peak in monthly geomagnetic index *aa* in pre-cycle (N-1) and the peak in the monthly sunspots numbers in the ascending phase of the subsequent solar cycle N. The peak in the monthly sunspots numbers is chosen before the rising slope of a second peak is deviated significantly from the generally rising slope of the selected peak in the ascending phase of the sunspot activity. The rising slope of the second peak is measured from the selected peak to the second peak (the intermediate dips are ignored). The peak in the monthly geomagnetic index *aa* in pre-cycle (N-1) is chosen previously as shown in Fig. 6. The average time delay as shown by the first curve is 57.6 ± 3.1 months.

Also appearing in Fig. 9 is a second curve which shows the number of months for the maximum averaged monthly sunspots number *Rz*(max) to appear after the solar cycle N has started. The average time delay is 49.1 ± 9.8 months.

The first curve, having a smaller varying amplitude, is statistically significant. It implies that the electromagnetic pole members which are emitting the electromagnetic signals at high latitude and received by the Earth, have been moving in each solar cycle, with a time-delayed time interval which is found to be statistically significant, toward the Sun’s Equator and to start forming the ascending phase in the sunspot’s butterfly pattern.

For example, two movable blocks *A*_−2_ and *B*_−2_ would sit between *A*_−3_ and *B*_o_ (or *A*_o_) at time, *t* = −2, in the movable divided-blocks diagram of Fig. 2. The positions of *A*_−2_ and *B*_−2_ indicate that the electromagnetic pole members in the movable blocks *A*_−2_ and *B*_−2_ are situated at high latitude of the Sun 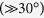. The movable blocks *A*_−2_ and *B*_−2_ would move downwardly along the line MEF toward the time *t* = 0 as time increases. They would respectively become *A*_o_ and *B*_o_ at time *t* = 0. The movable block *A*_−2_ would finally become *A*_3_ at *t* = 3 to form the ascending phase of the sunspot’s butterfly pattern. The interpretation on the movement of the electromagnetic pole members containing in the movable blocks of Fig. 2 is consistent with the results implied from the first curve of Fig. 9 as described above.

The time-delayed result on the rotational movement of the Sun’s magnetic poles confirms the observational conclusion made by Smith *et al.*^3^ that the Sun’s magnetic poles are moving toward the Equator during a solar cycle with the statistically evaluated time-delayed values as shown above. Furthermore, the first curve in Fig. 9 would allow for a reasonable prediction on the timing of the peak in the monthly sunspots numbers in the ascending phase of the next solar cycle.

The strong correlation and the similarity noted above have implied that a long-range force is in action, despite the appearance of randomness in the occurrence of the sunspots. The geomagnetic activity index *aa* in the late, and relatively quiet stage of the solar cycle could be contaminated by some mysterious events and unexpected solar flares, it is still an useful tool for use as a precursor signal to the upcoming solar cycle.

In summary, the strong correlation factors found and the consistency in the time-delayed values between the observed solar events and the received electromagnetic signals (the precursor signals) which have occurred many years earlier have implied a time-consuming and repeatable process. The electromagnetic pole members emitting the electromagnetic signals at the Sun’s high latitudes, as illustrated by the movable divided-blocks diagram, have migrated over time to the Sun’s Equatorial regions, where many visible sunspots would eventually appear (several years later).

### Method for Producing a Butterfly Pattern: a Preview

The sunspots, which often appear as a group, have been observed to occur in the Sun’s photosphere. The group may contain several, sometimes more than ten dark spots. The dark spots have been determined to be associated with a strong magnetic field. George Ellery Hale has linked the sunspots to the magnetic field in 1908. He has found the splitting of the sunspot’s spectral lines which are the typical signatures in the Zeeman Effect. The butterfly pattern formed by the sunspots during a regular 11–12 years cycle of a magnetic pole reversal was noted and published by E. Walter and Annie Maunder in 1904^29^.

A majority of the sunspots usually lasts for several days. This phenomenon indicates that the creation of a sunspot is a transient event. According to Equation (1), the North and South magnetic poles are precessing like a swing pendulum under the second-order electromagnetic torque *τ*_*x*_. A magnetic pole, “N” or “S”, is a distant region projected from a magnetic moment M onto the Sun’s photosphere.

At the end of a semi-cycle (one-half cycle) in a 2-dimensional precessing motion, the magnetic moment M rotates more slowly to come to a rest to reach one of its two terminating maximum angles before reversing the direction of its motion. The magnetic pole to which the magnetic moment M is projected would be instantaneously at rest at the end of every semi-cycle during the precession of the magnetic moment M. A prolonged stay of the projected magnetic pole member in a small region in the Sun would result in a perturbation that may eventually grow in size to become a visible sunspot we are seeing, when the magnetic moment M is momentarily at rest at one of its two terminating swinging angles.

The appearance of the sunspots and butterfly patterns over the years indicate that several magnetic moments may have some significant roles in the creation of a sunspot. The magnetic moments may be identified to come from different regions in the interior of the Sun. The Sun has a diameter of about 1.4 million km., or more than one hundred times of the Earth’s diameter. It would not be surprising if some of the magnetic moments may be attributable to a specific region in the Sun. The total magnetic moment, as we observed at a distance away from the Sun, is a vector-sum of all the magnetic moments attributable to the magnetic moments in various regions of the Sun.

Satellite measurements of the Earth’s magnetic field have indicated the existence of multiple magnetic moments. Olson^30^ has summarized the results discovered by Hulot *et al.*^31^ that two regions of reversed magnetic flux concentrated on the core-mantle boundary of the Earth have been identified. Also, as described in the section above, two precessing magnetic moments have been identified unambiguously in the pulsar PSR B1828–11^4^,^14^.

### Numerical calculation for a Butterfly Pattern

We consider a simple case when two magnetic moments are moving under the influence of the retardation electromagnetic torques. In this case, when a projected magnetic pole member from a first magnetic moment M is momentarily at rest at one of its two extreme end-points before reversing its motion, and a projected magnetic pole member from a second magnetic moment M′ comes relatively close, the combined magnetic field strength increases. A resulting perturbation due to the enhanced magnetic field strength and the rotational movement of the magnetic moments in the subsequent precessing cycles (with increasing polar angle *θ*) under the influence of the electromagnetic torques are found to have similarity to the apparent paths in the movement of the sunspots in a solar cycle. We present here the data to illustrate the viability of the method to produce a pattern similar to the sunspots butterfly pattern seen during a magnetic pole reversal.

Assume that the magnetic moment M is pointing in a x-y-z coordinate system having a set of values (x, y, z) = (rsin*θ*cos*ϕ*, rsin*θ*sin*ϕ*, rcos*θ*), and the angular momentum **L** is directed along the z-axis as shown in Fig. 1. The *y* − *z* plane is rotated about z-axis by *ϕ* − 90° so that the magnetic moment M lies in the *y*′*z* plane. We have, for a new *x*′ − *y*′ − *z*′ coordinate system,


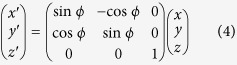


where *ϕ* is the azimuthal angle measured from the x-axis.

As the electromagnetic torque *τ*_*y*′_ is acting in the *y*′*z*′ plane, it causes the magnetic moment M to make an infinitely small change in the angle by Δ*γ*. After the rotation, the new values of (*x*′, *y*′, *z*′), as seen in the *x*′ − *y*′ − *z*′ coordinate system, would have a new set of values (*x*″, *y*″, *z*″) given by,


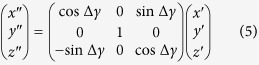


An inverse transformation of Equation (4) is made to calculate the new position of the magnetic moment M in the inertial coordinate system (*x*, *y*, *z*).

We note that the magnetic moment M would acquire an incremental velocity for each incremental value of the angle Δ*γ*. The velocity would eventually vary over time under the influence of the electromagnetic torque *τ*_*y*′_.

The infinitely small rotated angle Δ*γ* would carry the magnetic moment M out of the *y*′ − *z*′ plane. As a result, a new azimuthal angle *ϕ*′ must be calculated from the inverse transformation of Equation (4). The above procedure is repeated to obtain successive values in the positions of the magnetic moment M under the influence of the electromagnetic torques. The procedure above would also ensure that the new electromagnetic torque *τ*_*y*′_ is always acting along the new *y*′ axis to cause an infinitely small change in the corresponding angle away from the *y*′*z*′ plane. Similarly, the other electromagnetic torque *τ*_*x*′_ would always act along new *x*′ axis to cause a precession of the magnetic moment M as demanded by Equation (1), and the magnetic moment M is rotated as seen in the inertial *x* − *y* − *z* coordinate system.

**Figure 10:**
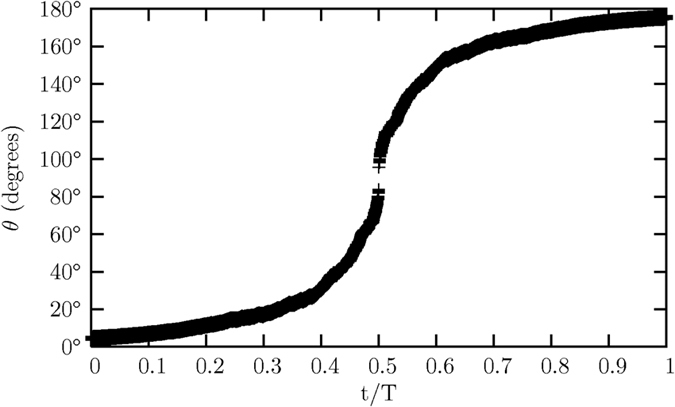
The polar angle *θ* of magnetic moment M increases with time *t*.

**Figure 11:**
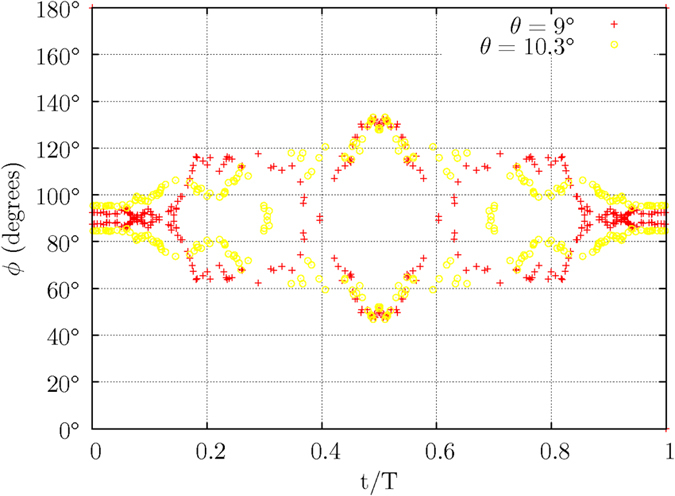
The azimuthal angles of two magnetic moments at their *θ*_*peak*_.

**Figure 12:**
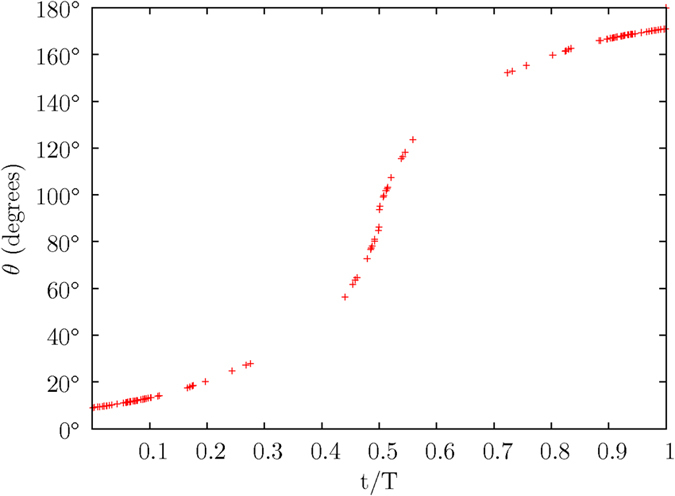
Values of polar angle *θ* when the projected magnetic pole members meet.

**Figure 13:**
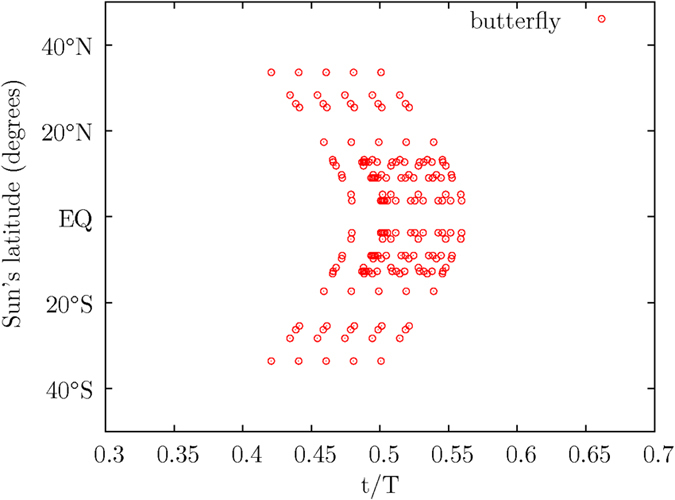
A butterfly pattern produced from projected magnetic pole members.

The numerical calculation is carried out according to the procedures laid out above. The results are shown in Figs (10, 11, 12, 13).

The magnetic moments employed in the numerical calculation are inclined at 9° and 10.3° relative to the Sun’s rotational axis. The azimuthal angles are respectively 90° and 95°. Both magnetic moments are executing a 2-dimensional precession under the second-order electromagnetic torque *τ*_*x*′_, while also undergoing at the same time a magnetic pole reversal caused by the third-order electromagnetic torque *τ*_*y*′_. Figure 10 shows the increasing value of the polar angle *θ* of the magnetic moment M having an initial inclined angle *θ*_o_ of 9°. The magnetic pole reversal occurs when the polarity of the magnetic moment M is eventually reversed at the end of a solar cycle, i.e. the magnetic moment M is pointing away in a direction opposite to its original direction. Figure 11 shows the variation in the azimuthal angles *ϕ* of the two magnetic moments, when their respective inclined angles *θ* are at their peaks in each cycle of a 2-dimensional precessing motion.

At the instantaneous terminating end points of each semi-cycle in a 2-dimensional precessing motion, each magnetic moment would cause a disturbance in the Sun, when the projected magnetic poles are moving at their slowest pace in a precessing cycle and are in a position about to reverse the direction of the motion at their end points. The combination of two projected magnetic poles pointing in about the same spot in the Sun at about the same time in our simple illustration here would result in a stronger perturbation in the Sun. The conditions imposed in this scheme for producing a visible turbulence in the Sun in Fig. 12 are, Δ*θ* = 0.2°, Δ*ϕ* = 3°, Δ*t* = 0.005*T*, where *T* is the period of a magnetic pole reversal.

As stated earlier, some of the magnetic moments may have different angles of inclination, the sunspots produced by projected magnetic poles would occur at different times. As shown in Fig. 2 above, a multiple sets of electromagnetic pole members in the movable blocks, for example, B_3_, would slide down along a line parallel to MEF. The block B_3_ would become B_6_, as the time *t* increases. For simplicity, the curve starting from about *θ* = 60° (Fig. 12), which corresponds to the Sun’s latitude at 30*N*, to *θ* = 90° (Sun’s Equator) is duplicated two times near the time *t* = 0.5 in Fig. 13. The duplicated curve is spaced apart at Δ*t* = 0.02*T* from the previous one, starting at *t* = 0.5. Due to the inversion symmetry, the South magnetic pole of the Sun would move upward during a magnetic pole reversal, while the North magnetic pole is moving downward from the North Hemisphere to the South Hemisphere, the data for the South magnetic pole is mirrored in Fig. 13.

As shown in Fig. 13, the pattern in the diagram visibly resembles the shape of a butterfly, which has been observed throughout the centuries. The slope shown in the upper left-most “left-leaning” curves in Fig. 13, which is seen to have a small curvature, is the path traced out by the moving magnetic pole of the magnetic moment M, when the magnetic pole is moving from the North to the Sun’s Equator. The “left-leaning” slope in Fig. 13 is consistent with the observed left-leaning paths seen in many well-known Sun’s butterfly patterns published in many popular articles^28^. The unique signature of these many left-leaning paths indicates that various magnetic moments are swinging and moving in groups across the Sun’s Equator at successive intervals of time.

The precessing motion of the magnetic moments M at their instantaneous terminating end points may cause a perturbation in the Sun when the magnetic moments are momentarily at rest before starting to swing again in the reversed direction. It is similar to a pendulum swinging to one of its extreme points before reversing its motion. The pendulum would spend more time there at its extreme points compared with all other points in its path. However, this unique characteristic in the rotational movement of the magnetic moment M would mean a stronger perturbation and a higher chance for producing a turbulence at the extreme end points of its precessing motion. The turbulence may grow in size to become a sunspot visible to us.

The distinct “left-leaning” paths in the observed butterfly pattern is produced unambiguously in Fig. 13 in the numerical calculation for the corrective motion of the magnetic moment M in a rotating body. The observed butterfly pattern is a consequence of the higher-orders correction to the rotational movement of the rotating magnetic moment M. It is a result combining from the precessing motion of the magnetic moments M under the second-order electromagnetic torque *τ*_*x*_ and the slower magnetic pole reversal motion under the third-order electromagnetic torque *τ*_*y*_ of Equation (2).

The calculated pattern, which is similar in shape, but differs in the distribution of data compared with the observed butterfly diagram, is to serve as an illustration. The method is presented for further discussion and improvement. It is noted that some of the sunspots in a smaller interaction region would usually multiply, leading to groups of evolving sunspots. Different magnetic pole members may not have the same strength in a solar cycle, especially during the initial and the final phase of the solar cycle.

The observed periodic reversal in the polarity of the magnetic poles in the Sun is caused by the apparent movement of the projected magnetic poles members under the influence of the third-order electromagnetic torque *τ*_*y*_. The North and South magnetic poles would swap their positions when each of them repeatedly moves across the Sun’s Equator from one hemisphere to another over time.

Due to the arithmetic trap which arises from the inclined angle of the magnetic moment M at *θ* = 90° from the *z*–axis, the numerical calculation is evaluated until the magnetic moment M approaches near the point at *θ* = 90°. The data in Figs (10, 11, 12, 13) are mirrored after *t* = 0.5.

### The Maunder Minimum and Other Cyclic Phenomenon

Besides the pair of magnetic moments illustrated above, other magnetic moments arising from a certain region in the interior of the Sun may form a group. One group of magnetic moments may differ from other groups in the strength and the spatial distribution of the magnetic moments. Some of the magnetic moments could have been shifted by turbulent shock waves and have resulted in an offsetting distance from the center of the Sun.

**Figure 14:**
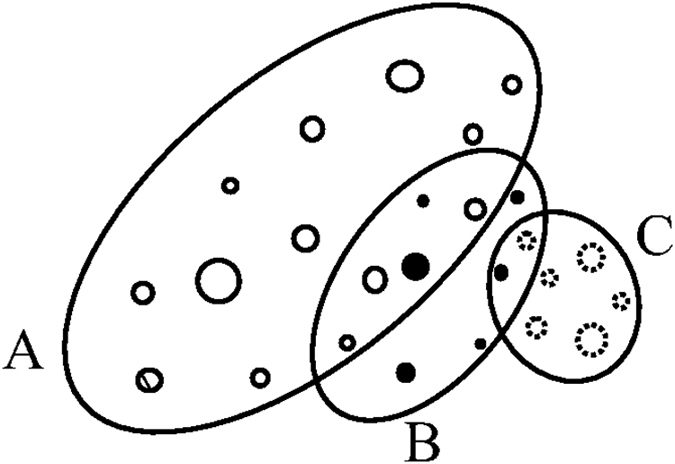
Members in the groups with differing magnetic field strengths.

We consider a group A, consisting of several magnetic moments, as shown in Fig. 14. The size of a circle in Fig. 14 represents the magnetic field strength of a specific magnetic moment in the group. Another group B, having a different spatial distribution (or locations) of magnetic moments and denoted by a number of dark circles, is moving relative to group A. To a first-order approximation, we consider that the group B is precessing about the group A in a relative periodic motion.

When the magnetic pole members, which are the projections from the magnetic moments in the groups, are approaching each other, the number of sunspots would increase. At a certain phase during the relative periodic movements between the groups of the magnetic moments, there is a chance that only a few of the projections from the magnetic moments would meet at the same place in the Sun while rotating. This would result in a very few sunspots. Such a phenomenon has been observed, notably the Maunder Minimum, when very few sunspots were noticed and recorded. The Maunder Minimum has been linked to the “Little Ice Age” in the 16th Century when the climate was unusually cold throughout Europe^32^.

Other methods using the cosmogenic isotopes 14C and 10Be have indicated that there are some systematic variations in the number of sunspots in the past 11,400 years^33^. The neutrons from the Sun arriving at the Earth are captured by nitrogen atoms in the Earth’s atmosphere and the isotope 14C is formed. By analyzing the isotopes 14C found in various natural stratified archives, such as tree rings or ice cores, the variation in the number of sunspots over time can be indirectly interpreted.

Several periods of low solar activity have been implied from the change in the carbon-14 record. The changes in the carbon-14 are not exclusively caused by the changes in the solar activity. It has however provided us with a picture on the past history of the solar activity of the Sun. The low solar activity in the past 1000 years had occurred on about AD 1645–1715 (Maunder Minimum), AD 1450–1550 (Spörer Minimum), ~AD 1330 (Wolf Minimum) and ~AD 1050 (Oort Minimum). They are approximately ~240 years apart^34^.

The North-South asymmetry of the sunspot activity has been reported^35^,^36^. One of the likely scenario is the offset of the magnetic moments from the center of the Sun. The offset of the major dipole moment also happens for the Earth’s magnetic dipole^37^.

A regular feature in the solar activity, with repeatable minima at various time, would favor a configuration in which two or more groups, each with a collection of a distributed magnetic moments similar to the one shown in Fig. 14, are moving in a relative motion with each other slowly over time.

## Discussion

The sunspots are seen to have randomly occurred in the Sun. However, their apparent migration paths toward the equatorial region of the Sun and the subsequent observation of the sunspot butterfly diagrams left behind in each of the solar cycles have strongly suggested a regular or periodic motion of a long-range force. Each solar cycle is also matching at the same time the reversal in the polarity of the magnetic poles of the Sun. Such a coincidence in these two distinct events is really intriguing and interesting.

The variation in the observed number of sunspots, which are often clustered in a group at various times, is an indication that points to a collection of distributed magnetic moments. A rather difficult task is the identification of different magnetic moments and their relative locations in the Sun.

The configuration in Fig. 14 shows a spatial distribution of the magnetic moments and their strengths, as the group B rotates with respect to group A. The configuration, when their orientations in a three dimensional space are included, would imply some variations in the observed solar features. The last solar cycle is the longest solar period of sunspots we have seen. The last solar cycle 23 has spanned about 12.5 years^38^, compared with other solar cycles having an average of 11–12 years in the past history. It is likely that not all of the group members would correspond to at least a member in another group in a specific solar cycle, when their projected magnetic pole members are moving to meet with each other to produce a visible sunspot. A member in a leading group in the beginning phase of a new solar cycle making an unexpected early encounter with a member of another group would alter the perceived period of a solar cycle. The period of the new solar cycle in this case would have been visibly lengthened.

One would wonder if the Sun would be much cooler, when the groups A and B specified in the above simple configuration would depart from each other for an extended time before coming back together again.

It is reasonable to conclude from the data analysis and the working mechanism found in the Precursor Method described above that the observed sunspots and the perturbation caused by the precessing magnetic moments at their instantaneous terminating end points in a precessing cycle are closely related. Another phenomenon closely related to the sunspots is the occurrences of solar flares near the sunspots. The rate of the solar flares also tends to reach a maximum activity level in a Solar Maxima when the sunspots are most active. It is likely due to two or more intense projected magnetic pole members which are approaching each other before merging. The swing speeds of the projected magnetic pole members in a two dimensional precessing motion would be measurable and predictable. A new focus on the precessing magnetic moments and their relative strengths is desirable. Their eventual identification in the Sun would yield many fruitful results in the future prediction on the activity of solar flares.

## Additional Information

**How to cite this article**: Ng, K. K. Prediction Methods in Solar Sunspots Cycles. *Sci. Rep.* **6**, 21028; doi: 10.1038/srep21028 (2016).
